# Supplementary material for: Clinical and biomarker analyses of sintilimab versus chemotherapy as second-line therapy for advanced or metastatic esophageal squamous cell carcinoma: a randomized, open-label phase 2 study (ORIENT-2)
Source: Nat Commun. 2022 Feb 14;13:857. doi: 10.1038/s41467-022-28408-3 (PMC8844279; doi:10.1038/s41467-022-28408-3)
Supplement: Supplementary file 1 — Supplementary Information [file 41467_2022_28408_MOESM1_ESM.pdf]

Clinical and biomarker analyses of sintilimab versus chemotherapy as second-line therapy for advanced or metastatic esophageal squamous cell carcinoma: a randomized, open-label phase 2 study (ORIENT-2)

Supplementary Appendix

Table of Contents

Supplementary Table 1. Survival analysis results of 28 immune cell signatures..............................3
Supplementary Table 2. Survival analysis results of 45 tumor signaling pathways..........................5
Supplementary Table 3. The P-value of different weight coefficient from Fleming-Harrington Test 8
Supplementary Table 4. The change of health-related quality of life of patients in FAS from baseline 9
Supplementary Table 5. The list of study sites...10
Supplementary Fig. 1 Forest plot for subgroup analyses of progression-free survival. 11
Supplementary Fig. 2 Kaplan-Meier plots of survival in different TCR clonality or mTBI subgroups of the sintilimab group. 12

Supplementary Table 1. Survival analysis results of 28 immune cell signatures

	OS						PFS					
	Sintilimab			Chemo			Sintilimab			Chemo		
	P-value	HR	95% CI	P-value	HR	95% CI	P-value	HR	95% CI	P-value	HR	95% CI
Activated B cell	0.669	1.13	0.64-1.99	0.347	1.33	0.74-2.39	0.030	0.54	0.31-0.94	0.415	1.30	0.69-2.46
Activated CD4 T cell	0.464	1.24	0.70-2.19	0.435	1.26	0.71-2.25	0.308	1.33	0.77-2.31	0.520	0.81	0.44-1.52
Activated CD8 T cell	0.798	1.08	0.61-1.89	0.303	1.36	0.76-2.43	0.285	0.74	0.42-1.29	0.760	0.91	0.48-1.71
Central memory CD4 T cell	0.337	1.32	0.75-2.33	0.390	0.77	0.43-1.39	0.455	0.81	0.47-1.40	0.416	0.77	0.40-1.46
Central memory CD8 T cell	0.773	0.92	0.52-1.62	0.888	1.04	0.59-1.86	0.407	0.80	0.46-1.37	0.960	0.98	0.52-1.88
Effector memeory CD4 T cell	0.684	0.89	0.5-1.57	0.344	1.33	0.74-2.39	0.146	0.66	0.38-1.15	0.467	0.79	0.42-1.49
Effector memeory CD8 T cell	0.912	1.03	0.59-1.81	0.954	0.98	0.55-1.75	0.458	0.81	0.47-1.40	0.929	0.97	0.52-1.81
Gamma delta T cell	0.990	1.00	0.57-1.76	0.544	0.84	0.47-1.49	0.449	0.81	0.47-1.40	0.782	1.09	0.58-2.04
Immature B cell	0.632	1.15	0.65-2.02	0.530	1.21	0.67-2.17	0.188	0.69	0.40-1.20	0.857	1.06	0.57-1.99
Memory B cell	0.147	1.53	0.86-2.70	0.326	1.34	0.75-2.40	0.852	0.95	0.55-1.63	0.260	0.70	0.37-1.31
Regulatory T cell	0.651	1.14	0.65-2.00	0.743	0.91	0.50-1.63	0.683	1.12	0.65-1.94	0.533	1.22	0.65-2.31
T follicular helper cell	0.255	0.72	0.41-1.27	0.890	1.04	0.59-1.85	0.007	0.46	0.26-0.80	0.892	1.04	0.56-1.96
Type 1 T helper cell	0.568	1.18	0.67-2.07	0.323	1.34	0.75-2.4	0.317	0.76	0.44-1.31	0.515	1.24	0.65-2.36
Type 17 T helper cell	0.628	1.15	0.65-2.02	0.976	0.99	0.56-1.77	0.680	1.12	0.65-1.93	0.939	0.98	0.52-1.83
Type 2 T helper cell	0.630	1.15	0.65-2.02	0.346	1.32	0.74-2.34	0.996	1.00	0.58-1.73	0.963	0.99	0.53-1.84
Activated dendritic cell	0.386	1.28	0.73-2.25	0.442	0.80	0.45-1.42	0.366	0.78	0.45-1.34	0.171	1.58	0.82-3.04
CD56bright natural killer cell	0.700	0.89	0.51-1.58	0.341	0.76	0.42-1.35	0.412	0.79	0.46-1.38	0.947	1.02	0.54-1.94
CD56dim natural killer cell	0.649	0.88	0.50-1.54	0.139	0.65	0.36-1.15	0.143	0.67	0.39-1.15	0.661	0.87	0.46-1.64
Eosinophil	0.125	1.56	0.88-2.74	0.512	1.21	0.68-2.17	0.096	1.60	0.92-2.77	0.902	1.04	0.55-1.96
Immature dendritic cell	0.409	1.27	0.72-2.25	0.041	0.54	0.30-0.98	0.078	0.61	0.35-1.06	0.598	1.18	0.63-2.22

Macrophage	0.473	1.23	0.70-2.17	0.468	1.24	0.70-2.20	0.349	1.30	0.75-2.23	0.987	1.01	0.53-1.91
Mast cell	0.955	0.98	0.55-1.75	0.187	1.50	0.82-2.76	0.568	0.85	0.49-1.49	0.321	1.42	0.71-2.82
MDSC	0.572	1.18	0.67-2.07	0.681	1.13	0.64-2.00	0.886	0.96	0.56-1.65	0.842	1.07	0.57-1.99
Monocyte	0.321	1.33	0.76-2.33	0.586	0.85	0.46-1.55	0.778	1.08	0.63-1.87	0.834	1.07	0.56-2.04
Natural killer cell	0.303	1.35	0.76-2.39	0.573	1.19	0.66-2.14	0.762	0.92	0.53-1.58	0.578	1.20	0.64-2.24
Natural killer T cell	0.672	0.89	0.50-1.56	0.181	1.48	0.83-2.63	0.812	0.94	0.54-1.61	0.561	1.21	0.64-2.29
Neutrophil	0.304	1.35	0.76-2.40	0.718	0.89	0.49-1.64	0.127	0.65	0.38-1.13	0.580	1.19	0.64-2.24
Plasmacytoid dendritic cell	0.664	1.13	0.64-2.00	1.000	1.00	0.56-1.78	0.383	0.79	0.46-1.35	0.201	1.51	0.80-2.84

P values were based on a two-sided Wald test. Source data are provided as a Source Data file.

Supplementary Table 2. Survival analysis results of 45 tumor signaling pathways

	OS						PFS					
	Sintilimab			Chemo			Sintilimab			Chemo		
	P-value	HR	95% CI	P-value	HR	95% CI	P-value	HR	95% CI	P-value	HR	95% CI
Androgen Signaling	0.651	1.14	0.65-2.01	0.487	1.23	0.69-2.20	0.901	0.97	0.56-1.67	0.610	0.85	0.45-1.59
Antigen Presentation	0.373	1.29	0.74-2.27	0.212	0.69	0.39-1.23	0.551	1.18	0.68-2.05	0.858	1.06	0.57-1.98
Apoptosis	0.952	1.02	0.58-1.79	0.755	1.10	0.61-1.97	0.844	0.95	0.55-1.63	0.993	1.00	0.54-1.88
Autophagy	0.906	1.03	0.59-1.83	0.164	0.66	0.36-1.19	0.868	1.05	0.61-1.81	0.561	0.83	0.44-1.56
Cell Adhesion & Motility	0.888	1.04	0.59-1.83	0.309	1.35	0.76-2.40	0.860	1.05	0.61-1.81	0.987	1.01	0.54-1.88
Cell Cycle	0.478	0.81	0.46-1.44	0.595	1.17	0.66-2.08	0.634	1.14	0.66-1.96	0.591	0.84	0.45-1.58
Chemokine Signaling	0.676	0.89	0.50-1.56	0.618	0.86	0.48-1.54	0.408	0.79	0.46-1.37	0.496	1.25	0.66-2.39
Cytotoxicity	0.509	0.82	0.46-1.47	0.204	1.49	0.81-2.74	0.192	0.68	0.39-1.21	0.690	1.14	0.59-2.20
DNA Damage Repair	0.772	0.92	0.52-1.62	0.705	1.12	0.63-2.00	0.934	1.02	0.59-1.77	0.531	1.22	0.65-2.28
ECM Remodeling & Metastasis	0.797	1.08	0.61-1.89	0.233	1.42	0.80-2.53	0.794	1.07	0.62-1.85	0.918	1.03	0.55-1.93
EGFR Signaling	0.544	0.84	0.48-1.48	0.715	0.90	0.50-1.61	0.327	0.76	0.44-1.32	0.685	0.88	0.46-1.66
EMT	0.296	1.35	0.77-2.39	0.621	0.86	0.48-1.56	0.698	1.11	0.65-1.91	0.285	0.71	0.37-1.34
Epigenetic & Transcriptional Regulation	0.870	1.05	0.60-1.84	0.977	1.01	0.56-1.81	0.608	0.87	0.50-1.50	0.034	0.47	0.23-0.94
ERBB2 Signaling	0.362	0.77	0.44-1.35	0.978	1.01	0.57-1.80	0.384	0.79	0.46-1.35	0.631	0.86	0.45-1.61
Estrogen Signaling	0.390	1.29	0.72-2.28	0.478	0.81	0.44-1.46	0.727	0.91	0.53-1.56	0.393	0.75	0.39-1.44
FGFR Signaling	0.735	0.91	0.52-1.59	0.619	0.86	0.49-1.53	0.576	0.86	0.49-1.48	0.538	1.22	0.65-2.29
Glucose Metabolism	0.716	1.11	0.63-1.95	0.032	0.51	0.28-0.95	0.793	1.08	0.63-1.85	0.132	0.61	0.32-1.16
Glutamine Metabolism	0.709	0.89	0.49-1.62	0.336	1.34	0.74-2.41	0.771	0.92	0.54-1.59	0.190	0.64	0.33-1.25
Hedgehog	0.914	1.03	0.59-1.81	0.743	0.91	0.51-1.62	0.262	1.37	0.79-2.35	0.965	1.01	0.54-1.92
HIF1 Signaling	0.038	1.86	1.04-3.34	0.025	0.49	0.26-0.92	0.865	1.05	0.60-1.82	0.247	0.68	0.35-1.31

Hippo Signaling	0.857	1.05	0.60-1.85	0.151	0.63	0.34-1.18	0.979	0.99	0.58-1.71	0.134	0.60	0.31-1.17
Immortality & Stemness	0.856	0.95	0.53-1.68	0.613	1.16	0.65-2.07	0.844	1.06	0.61-1.81	0.922	1.03	0.55-1.93
Inflammation	0.467	1.24	0.70-2.18	0.487	1.23	0.69-2.18	0.900	0.97	0.56-1.66	0.879	1.05	0.56-1.97
Interferon Response	0.795	0.93	0.53-1.63	0.061	0.57	0.31-1.03	0.848	0.95	0.55-1.63	0.643	1.16	0.61-2.20
Interleukin Signaling	0.310	0.75	0.42-1.31	0.066	1.74	0.96-3.13	0.935	0.98	0.57-1.68	0.848	0.94	0.50-1.76
JAK-STAT Signaling	0.597	0.86	0.49-1.51	0.923	0.97	0.54-1.75	0.957	1.02	0.59-1.74	0.708	0.89	0.47-1.66
Lipid Metabolism	0.225	0.70	0.40-1.24	0.789	0.92	0.52-1.65	0.313	1.33	0.77-2.30	0.430	0.78	0.41-1.46
MAPK Signaling	0.323	1.33	0.75-2.37	0.743	0.90	0.50-1.65	0.630	0.87	0.51-1.51	0.952	0.98	0.52-1.86
MET Signaling	0.017	2.04	1.14-3.66	0.078	1.67	0.94-2.97	0.104	1.58	0.91-2.75	0.876	0.95	0.50-1.81
mTOR Signaling	0.324	1.33	0.75-2.34	0.675	0.88	0.49-1.59	0.504	1.20	0.70-2.07	0.250	0.69	0.36-1.30
Myc	0.887	0.96	0.54-1.69	0.080	0.58	0.31-1.07	0.346	1.30	0.75-2.25	0.373	0.75	0.40-1.41
Myeloid Immune Evasion	0.427	0.79	0.45-1.41	0.064	1.78	0.97-3.27	0.075	0.60	0.34-1.05	0.827	1.07	0.57-2.04
NF-kB Signaling	0.850	0.95	0.54-1.67	0.395	1.28	0.72-2.28	0.993	1.00	0.58-1.72	0.804	1.08	0.58-2.03
Notch Signaling	0.491	0.82	0.46-1.45	0.162	0.65	0.36-1.19	0.242	0.72	0.41-1.25	0.573	0.83	0.44-1.58
Nrf2 & Oxidative Stress	0.130	0.64	0.36-1.14	0.146	0.64	0.35-1.17	0.016	0.50	0.28-0.88	0.962	0.98	0.52-1.86
p53 Signaling	0.796	1.08	0.61-1.90	0.598	1.18	0.64-2.15	0.400	1.26	0.73-2.18	0.132	0.60	0.31-1.17
PDGF Signaling	0.708	1.11	0.63-1.95	0.906	1.04	0.57-1.87	0.938	1.02	0.59-1.75	0.770	1.10	0.59-2.06
PI3K-Akt Signaling	0.034	1.84	1.05-3.23	0.757	0.91	0.51-1.63	0.469	1.23	0.71-2.13	0.303	0.71	0.37-1.36
Senescence	0.696	1.12	0.63-1.98	0.189	0.67	0.37-1.22	0.681	0.89	0.52-1.54	0.821	1.07	0.58-2.01
TCR Signaling	0.769	1.09	0.62-1.92	0.953	0.98	0.55-1.76	0.826	0.94	0.55-1.62	0.947	0.98	0.52-1.83
TGF-beta Signaling	0.319	0.75	0.43-1.32	0.741	1.10	0.61-1.99	0.798	0.93	0.54-1.60	0.411	0.77	0.40-1.45
TNF Superfamily	0.694	0.89	0.51-1.57	0.319	1.34	0.75-2.41	0.289	1.35	0.78-2.34	0.905	1.04	0.55-1.96
Tumor Antigen	0.556	0.84	0.48-1.49	0.182	0.67	0.38-1.20	0.542	0.84	0.48-1.48	0.056	1.93	0.98-3.79
VEGF Signaling	0.801	0.93	0.52-1.65	0.171	0.65	0.35-1.20	0.247	0.72	0.42-1.25	0.558	0.83	0.44-1.56
Wnt Signaling	0.011	0.46	0.25-0.84	0.513	1.21	0.68-2.17	0.677	0.89	0.52-1.54	0.275	0.69	0.35-1.34

P values were based on a two-sided Wald test. Source data are provided as a Source Data file.

Supplementary Table 3. The P-value of different weight coefficient from Fleming-Harrington Test

Different Weight coefficient	P-value (two-sided)
FH(0,0.2)	0.00823
FH(0,0.5)	0.00370
FH(0,1)	0.00227
FH(1,0)	0.24214

Supplementary Table 4. The change of health-related quality of life of patients in FAS from baseline

	**Sintilimab (N=95)**				**Chemo (N=95)**			
Time	N	EQ-5D-5L VAS Mean (SD)	EQ-5D-5L Index Mean (SD)	EORTC QLQ-C30 General health Mean (SD)	N	EQ-5D-5L VAS Mean (SD)	EQ-5D-5L Index Mean (SD)	EORTC QLQ-C30 General health Mean (SD)
Baseline	87				84			
Week 6	70	-1.81 (15.7186)	-0.03 (0.1717)	-0.11 (22.9036)	58	-6.54 (14.2510)	-0.06 (0.1623)	-3.06 (22.7037)
Week 12	52	-3.52 (13.4288)	-0.03 (0.1836)	3.24 (17.1637)	28	-6.30 (15.6911)	-0.13 (0.1215)	-3.89 (24.7336)
Week 18	35	-5.23 (15.9154)	-0.04 (0.1494)	3.01 (21.0020)	9	2.00 (16.1245)	-0.15 (0.1776)	5.30 (29.4092)
Week 24	22	0.05 (12.5905)	0.01 (0.1268)	6.88 (14.1359)	6	4.50 (17.2366)	-0.07 (0.1361)	11.46 (23.5439)
Week 33	21	-2.05 (12.8549)	-0.03 (0.1445)	-0.76 (22.4063)	3	-3.33 (11.5470)	-0.19 (0.3349)	-2.78 (29.2657)
Week 42	17	-1.24 (16.2386)	0.00 (0.1569)	3.70 (23.9523)	1	0.00 (NA)	0.00 (NA)	25.00 (NA)

EQ-5D-5L: European Quality of Life-5 Dimensions; VAS: visual analogue scale; EORTC QLQ-C30: European Organisation for Research and Treatment of Cancer Quality-of-Life Questionnaire-Core 30. NA, not available.

Supplementary Table 5. The list of study sites

Site No.	Site	Enrollment Number
01	The fifth medical center of the PLA general hospital	18
02	Cancer hospital, Chinese academy of medical sciences	8
03	Peking Union Medical College Hospital，Chinese Academy of Medical Sciences	2
04	The first Affiliated Hospital of Zhejiang University	8
05	First Affiliated Hospital of Xi’an Jiaotong University	8
06	Harbin Medical University Cancer Hospital	4
09	Union Hospital affiliated to Tongji Medical College of Huazhong University of Science and Technology	5
10	The First Affiliated Hospital of Zhengzhou University	19
11	JIANGSU PROVINCE HOSPITAL	19
12	Xiangya Hospital Central South University	4
13	The first Bethune Hospital of Jilin University	3
14	The first affiliated hospital of Xiamen University	4
15	Zhejiang Cancer Hospital	3
16	Chinese People's Liberation Army General Hospital	3
17	Qilu Hospital of Shandong University	9
18	Guangdong Provincial People's Hospital	3
20	Henan Cancer Hospital	7
21	The first Hospital of China Medical University	6
22	Nanfang Hospital Southerng Medical University	1
23	Yunnan Cancer Hospital	2
25	The First Affiliated Hospital of Anhui Medical College	10
26	Hunan Cancer Hospital	1
27	The first affiliated hospital of soochow university	10
28	The first affiliated hospital of Bengbu Medical College	4
29	Fujian Provincial Hospital	11
30	900 Hospital of the Joint Logistics Team	1
31	Liaoning Cancer Hospital	3
34	Gansu Provincial Cancer Hospital	1
38	Affiliated Hospital of Jining Medical University	1
39	Nantong Tumor Hospital	12

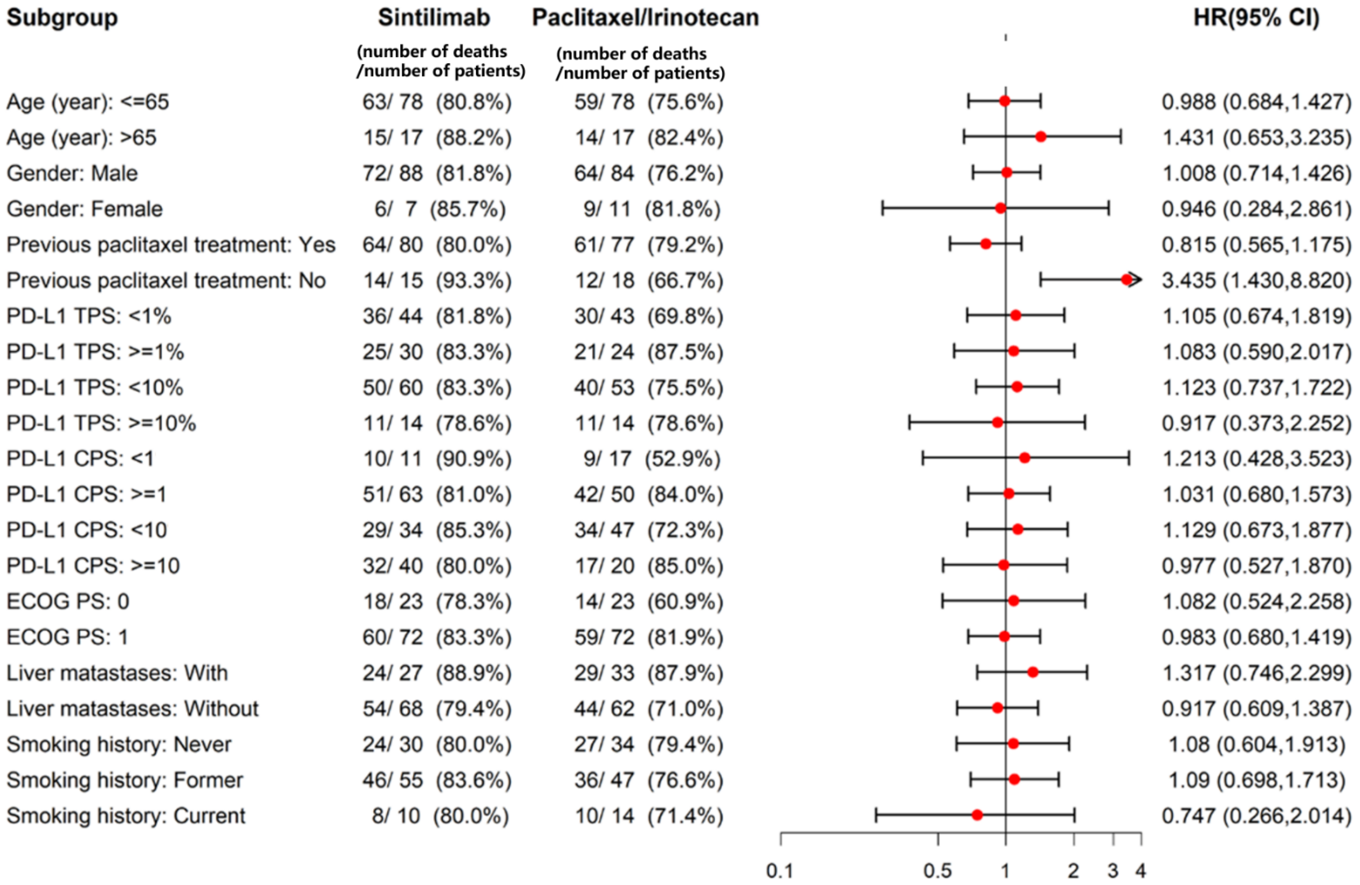

Supplementary Fig. 1 Forest plot for subgroup analyses of progression-free survival. Dots represent the cohort-specific hazards ratios with error bars corresponding to 95% CI bounds, which were calculated by using the univariate Cox regression model. *ECOG PS* Eastern Cooperative Oncology Group performance status, *HR* hazard ratio, *CI* confidence interval.

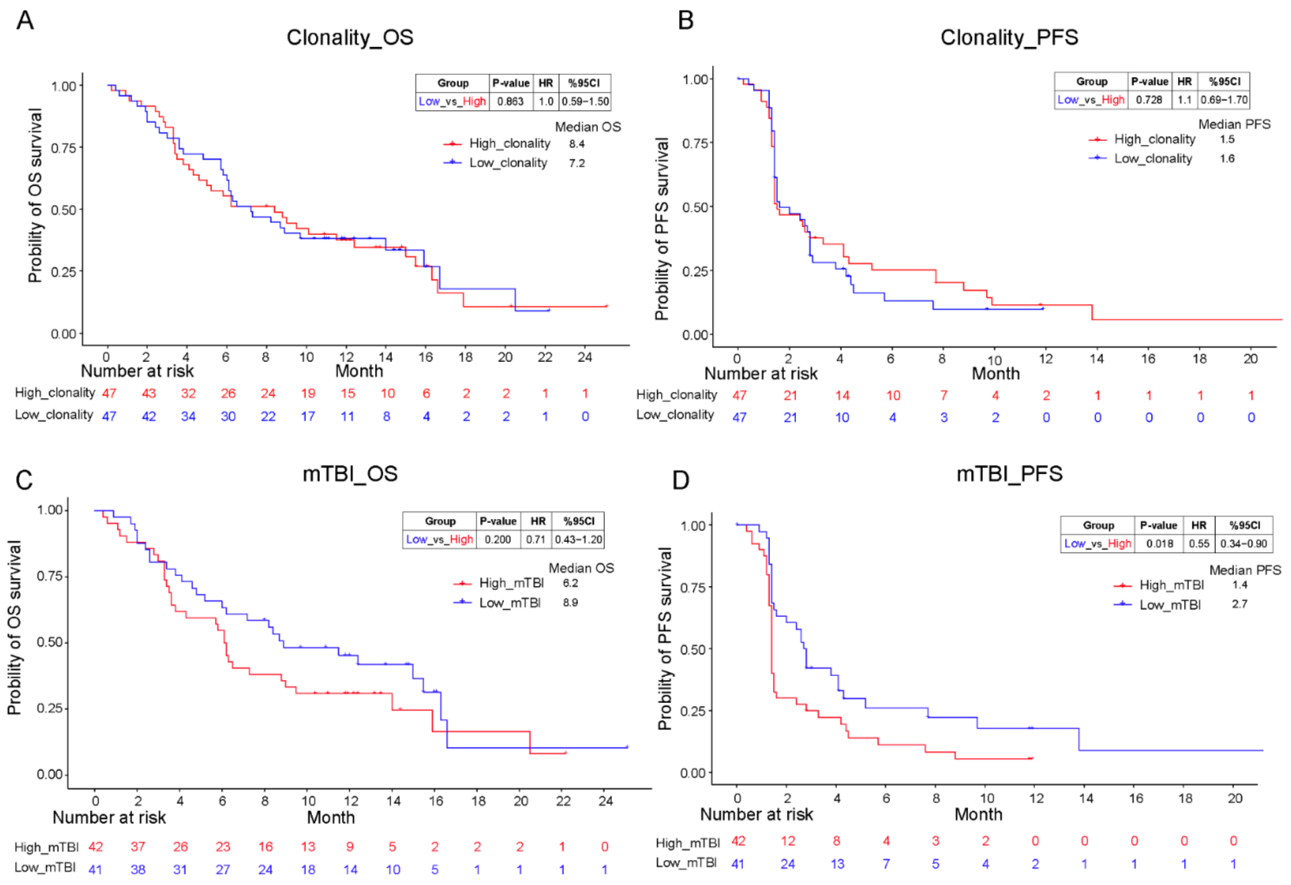

Supplementary Fig. 2 Kaplan-Meier plots of survival in different TCR clonality or mTBI subgroups of the sintilimab group. a, Overall survival in high and low TCR clonality subgroups; **b**, Progression free survival in high and low TCR clonality subgroups; **c**, Overall survival in high and low mTBI subgroups; **d**, Progression free survival in high and low mTBI subgroups. The high and low level groups of TCR clonality or mTBI are split by respective median value. P values were based on a two-sided Wald test. Source data are provided as a Source Data file.
