# Supplementary material for: Clinical and biomarker analyses of sintilimab versus chemotherapy as second-line therapy for advanced or metastatic esophageal squamous cell carcinoma: a randomized, open-label phase 2 study (ORIENT-2)
Source: Nat Commun. 2022 Feb 14;13:857. doi: 10.1038/s41467-022-28408-3 (PMC8844279; doi:10.1038/s41467-022-28408-3)
Supplement: Supplementary file 3 — Reporting Summary [file 41467_2022_28408_MOESM3_ESM.pdf]

nature portfolio

Corresponding author(s): Jianming Xu

Last updated by author(s): Jan 12, 2022

Reporting Summary

Nature Portfolio wishes to improve the reproducibility of the work that we publish. This form provides structure for consistency and transparency in reporting. For further information on Nature Portfolio policies, see our Editorial Policies and the Editorial Policy Checklist.

Statistics

For all statistical analyses, confirm that the following items are present in the figure legend, table legend, main text, or Methods section.

n/a	Confirmed	
☐	☒	The exact sample size (*n*) for each experimental group/condition, given as a discrete number and unit of measurement
☐	☒	A statement on whether measurements were taken from distinct samples or whether the same sample was measured repeatedly
☐	☒	The statistical test(s) used AND whether they are one- or two-sided<br>*Only common tests should be described solely by name; describe more complex techniques in the Methods section.*
☐	☒	A description of all covariates tested
☐	☒	A description of any assumptions or corrections, such as tests of normality and adjustment for multiple comparisons
☐	☒	A full description of the statistical parameters including central tendency (e.g. means) or other basic estimates (e.g. regression coefficient) AND variation (e.g. standard deviation) or associated estimates of uncertainty (e.g. confidence intervals)
☐	☒	For null hypothesis testing, the test statistic (e.g. *F*, *t*, *r*) with confidence intervals, effect sizes, degrees of freedom and *P* value noted<br>*Give P values as exact values whenever suitable.*
☒	☐	For Bayesian analysis, information on the choice of priors and Markov chain Monte Carlo settings
☒	☐	For hierarchical and complex designs, identification of the appropriate level for tests and full reporting of outcomes
☐	☒	Estimates of effect sizes (e.g. Cohen's *d*, Pearson's *r*), indicating how they were calculated

Our web collection on statistics for biologists contains articles on many of the points above.

Software and code

Policy information about availability of computer code

Data collection	No software was used to collect these data.
Data analysis	Statistical analyses for the clinical part were performed by using SAS version 9.4 (Cary,North Carolina, US). RNAseq analysis was performed by using FastQC(version 0.11.8), Trimmomatic,(version 0.36), STAR(version 2.7.0a), HTSeq(version 0.11.4) , R Survival package(version 2.44-1.1). CtDNA analysis was performed by using BWA(version 0.7.17), GATK(version 4.1.4.1), PyClone(version 0.13.1). TCR analysis was performed by using Pear and MiXCR(version 3.0.4).

For manuscripts utilizing custom algorithms or software that are central to the research but not yet described in published literature, software must be made available to editors and reviewers. We strongly encourage code deposition in a community repository (e.g. GitHub). See the Nature Portfolio guidelines for submitting code & software for further information.

Data

Policy information about availability of data

All manuscripts must include a data availability statement. This statement should provide the following information, where applicable:

- Accession codes, unique identifiers, or web links for publicly available datasets
- A description of any restrictions on data availability
- For clinical datasets or third party data, please ensure that the statement adheres to our policy

All study data are presented in the manuscript and supplementary files. Source data are provided with this paper, including the baseline patient information, the results of efficacy in all patients, the NLR, the results of RNA-sequencing, TCR clonality and molecular tumor burden index (mTBI) in the sintilimab group. The raw RNA-seq, TCR-seq and ctDNA-seq reads generated in this paper are deposited in Genome Sequence Archive (GSA) under the accession code HRA001773. The raw sequence data are available under restricted access because of data privacy laws.

Field-specific reporting

Please select the one below that is the best fit for your research. If you are not sure, read the appropriate sections before making your selection.

☒ Life sciences ☐ Behavioural & social sciences ☐ Ecological, evolutionary & environmental sciences

For a reference copy of the document with all sections, see nature.com/documents/nr-reporting-summary-flat.pdf

Life sciences study design

All studies must disclose on these points even when the disclosure is negative.

Sample size	It was estimated that the required death event number was 142 with an expected hazard ratio (HR) for OS of 0.7 ($\alpha = 0.2$, two-sided) under a statistical power of 80% and 180 patients were required with 90 patients in each group. Actually 190 patients were enrolled.
Data exclusions	Exclusion criteria was established in study protocol before the start of enrollment. All drop-out cases were described in CONSORT diagram and in manuscript. No other data was excluded from analysis.
Replication	This study is a phase 2 clinical study. Results can not be reproduced in this study. And all the described results were tested for replication by two independent statisticians.
Randomization	Patients were randomly (1:1) assigned to receive either sintilimab or the investigator's choice of chemo (paclitaxel or irinotecan), using an interactive web response system with a block size of a mixture of 2, 4, and 6, and with the stratification factor of the ECOG PS score (0 vs. 1).
Blinding	This is an open label study.

Reporting for specific materials, systems and methods

We require information from authors about some types of materials, experimental systems and methods used in many studies. Here, indicate whether each material, system or method listed is relevant to your study. If you are not sure if a list item applies to your research, read the appropriate section before selecting a response.

Materials & experimental systems

n/a	Involved in the study	
☒	☐	Antibodies
☒	☐	Eukaryotic cell lines
☒	☐	Palaeontology and archaeology
☒	☐	Animals and other organisms
☐	☒	Human research participants
☐	☒	Clinical data
☒	☐	Dual use research of concern

Methods

n/a	Involved in the study	
☒	☐	ChIP-seq
☒	☐	Flow cytometry
☒	☐	MRI-based neuroimaging

Human research participants

Policy information about studies involving human research participants

Population characteristics	Patients with histopathologically or cytologically confirmed locally advanced or metastatic ESCC aged between 18 and 75 years old were enrolled including male and female. Major eligibility criteria were at least one measurable lesion per Response Evaluation Criteria in Solid Tumors (RECIST), version 1.1, Eastern Cooperative Oncology Group (ECOG) performance status (PS) of 0 or 1, eligible to provide a fresh or archived tumor sample, radiological or clinical evidence of disease progression during or after first-line chemotherapy. Patients were excluded, who received prior PD-1 or PD-L1 inhibitors or received any radiotherapy, immunosuppressive drugs or the study drug within 4 weeks prior to the first drug dose.
Recruitment	Any patients with disease progression ESCC after first-line treatment, who was admitted at the center and met the criteria for inclusion/exclusion, was considered for recruitment. Patients were screened and the principal investigators were responsible for the evaluation and enrollment. Patients were recruited from 30 hospitals in China. Therefore, to the best of our knowledge, there was no selection bias during recruitment.Patients were randomly (1:1) assigned to receive either sintilimab or the investigator's choice of chemo (paclitaxel or irinotecan).
Ethics oversight	Study was approved by ethics committee of all hospitals (The fifth medical center of the PLA general hospital; Cancer hospital, Chinese academy of medical sciences; Peking Union Medical College Hospital, Chinese Academy of Medical Sciences; The first Affiliated Hospital of Zhejiang University; First Affiliated Hospital of Xi'an Jiaotong University; Harbin Medical University Cancer Hospital; Union Hospital affiliated to Tongji Medical College of Huazhong University of Science and Technology; The First Affiliated Hospital of Zhengzhou University; JIANGSU PROVINCE HOSPITAL; Xiangya Hospital Central South University; The first Bethune Hospital of Jilin University; The first affiliated hospital of Xiamen University; Zhejiang Cancer Hospital; Chinese People's Liberation Army General Hospital; Qilu Hospital of Shandong University; Guangdong Provincial People's Hospital; Henan Cancer Hospital; The first Hospital of China Medical University; Nanfang Hospital Southerng Medical University; Yunnan Cancer Hospital; The First Affiliated Hospital of Anhui Medical College; Hunan Cancer Hospital; The first affiliated hospital of soochow university; The first affiliated hospital of Bengbu Medical College; Fujian Provincial Hospital; 900 Hospital of the Joint Logistics Team; Liaoning Cancer Hospital; Gansu Provincial Cancer Hospital; Affiliated Hospital of Jining Medical University; Nantong Tumor Hospital). All the patients provided written informed consent before screening and we have complied with all relevant ethical regulations. The study was performed in accordance with ethical principles that have their origin in the Declaration of Helsinki and are consistent with ICH/Good Clinical Practice.

Note that full information on the approval of the study protocol must also be provided in the manuscript.

Clinical data

Policy information about clinical studies

All manuscripts should comply with the ICMJEguidelines for publication of clinical research and a completedCONSORT checklist must be included with all submissions.

Clinical trial registration	NCT03116152
Study protocol	The protocol included with submission.
Data collection	Between May 16, 2017 and August 30, 2018, 190 patients from 35 hospitals in China were enrolled in this study. And the data was cut off on August 2, 2019.
Outcomes	The primary endpoint was OS, defined as the time from randomization until death. Secondary endpoints were progression-free survival (PFS), which was the time from randomization to initial disease progression or death, evaluated by the investigator per RECIST v1.1; objective response rate (ORR), defined as the percentage of patients achieving complete response (CR) or partial response (PR) evaluated by the investigator per RECIST v1.1; disease control rate (DCR), which was the percentage of patients with the best overall response of CR, PR, or stable disease (SD) evaluated by the investigator per RECIST v1.1; time to response (TTR), which was the time from randomization to the time point of CR or PR; and duration of response (DOR),which was the time from the time point of CR or PR to initial disease progression or death.
